# Effect of Transarterial Chemoembolization on Health-Related Quality of Life in Patients With Hepatocellular Carcinoma

**DOI:** 10.7759/cureus.82531

**Published:** 2025-04-18

**Authors:** Susan George, Krishnadas Devadas, Prasanth Thayyil Sudheendran, Srijaya Sreesh, Shabnam Safeer, Jesse Jacob Skariah, Ann Mary George, Akhil Njamelil Visruthakumar, Gayathri Sivakumar, Minu Sajeev Kumar

**Affiliations:** 1 Gastroenterology and Hepatology, Government Medical College Thiruvananthapuram, Thiruvananthapuram, IND

**Keywords:** hcc, hrqol, liver cancer, qol, tace

## Abstract

Background and aim: We evaluated health-related quality of life (HRQoL) in patients with hepatocellular carcinoma (HCC) before and after transarterial chemoembolization (TACE) and the clinical and biochemical factors that can predict changes in quality of life (QoL).

Methods: A total of 45 patients were enrolled in the study and followed up for a period of three months. HRQoL was assessed using an HCC-specific questionnaire at baseline, two weeks, and three months. Tumor response was evaluated at six weeks using the modified Response Evaluation Criteria in Solid Tumors (mRECIST) criteria.

Results: Before TACE, the aspects of the functional scales most significantly impacted were global health status (53.1±29.3%) and physical functioning (68.7±20.7%). The commonly reported symptoms and their corresponding QoL scores were as follows: fatigue (46.4±32.5%), followed by insomnia (32.6±36.6%) and abdominal pain (32.2±30.8%). Financial constraints (63.0±37.1%) were also a significant concern for the patients. Most functional and symptom scores showed a reduction at two weeks and improvement at three months. Global health status improved to 68±28.2% (p=0.455), whereas physical functioning improved to 73.8±23.2% (p=0.005) at three months following TACE. After three months following TACE, the QoL scores for the following symptoms improved from baseline: fatigue (30.5±28.2; p=0.012), insomnia (25.9±27.4; p=0.896), and pain (28.5±30.8; p=0.005). High alpha-fetoprotein (AFP), high C-reactive protein (CRP), low albumin, and an increase in Model for End-stage Liver Disease (MELD) and Child-Turcotte-Pugh (CTP) scores were found to have a negative impact at three months.

Following TACE, 26 (57%), nine (20%), three (7%), and seven (16%) patients had complete response (CR), partial response (PR), stable disease (SD), and progressive disease (PD), respectively. Quality of life scores showed a positive response at three months in subjects with CR, PR, and SD, while in those with PD, QoL continued to deteriorate over time.

Conclusion: Effective symptom management, along with the implementation of coping strategies to improve functionality, is crucial when caring for patients who have undergone TACE, particularly during the first two weeks post-procedure.

## Introduction

Hepatocellular carcinoma (HCC) is the most common primary liver malignancy. The incidence of HCC in cirrhosis in India is 1.6% per year, with a preponderance in males (M:F=4:1) [1]. Non-alcoholic fatty liver disease (NAFLD) has emerged as the leading cause of cirrhosis, progressing to HCC. Hepatocellular carcinoma is diagnosed primarily with a contrast-enhanced CT and/or MRI scan of the abdomen using the Liver Imaging Reporting and Data System (LIRADS) staging system. LIRADS 5 is diagnostic of HCC [2]. The current management strategies for HCC are based on the widely used Barcelona Clinic Liver Cancer (BCLC) staging system. The 2022 BCLC update utilized tumor burden, presence of portal hypertension, liver function, and Eastern Cooperative Oncology Group (ECOG) performance for classifying HCC. For intermediate HCC, TACE is the most widely accepted treatment modality. For BCLC B subtype B2, TACE is the first-line therapy. TACE can also be performed for BCLC 0, BCLC A, and BCLC B1 stages when other therapeutic modalities are not expedient [3,4].

Transcatheter arterial chemoembolization (TACE), also known as transarterial chemoembolization, is a minimally invasive method of administering chemotherapy directly to a liver tumor via a catheter under digital subtraction angiography (DSA). The chemoembolic agent may be delivered as a mixture with Lipiodol (conventional TACE) or as an injection of drug-eluting beads (DEB-TACE). Over 95% of the blood supply to a liver tumor is from the hepatic artery. Therefore, embolization of the hepatic artery is performed to induce tumor necrosis while preserving background liver function. Doxorubicin is the most commonly used chemotherapeutic agent worldwide. The chemotherapy agent is mixed with iodized oil, typically Lipiodol. In conventional TACE, the administration of the chemotherapy agent is followed by mechanical embolization, typically using gel foam or polyvinyl alcohol (PVA) particles [5-7]. Tumor response to TACE is assessed using the modified Response Evaluation Criteria in Solid Tumors (mRECIST) criteria, which are classified into the following four categories: complete response (CR), partial response (PR), progressive disease (PD), and stable disease (SD).

Less than 20% of the patient population qualifies for curative treatment at the time of HCC diagnosis [6]. Palliation is offered to the majority of patients because of late-stage presentation, associated hepatic dysfunction, multiple comorbidities, and limited donor liver availability. Palliative therapy aimed to provide symptomatic relief, prolong survival, and improve quality of life (QoL). Palliative treatments can potentially have a detrimental effect on quality of life, mainly if complications arise. A decline in quality of life post-treatment can deter patients from pursuing and adhering to future treatments. TACE enhances the QoL of HCC patients probably by palliating most of the disturbing symptoms such as itching, lethargy, sleep disorders, sexual dysfunction, and abdominal discomfort [8].

This study aimed to evaluate the changes in health-related quality of life (HRQoL) in patients with HCC before and after TACE. Also, we tried to identify the clinical and biochemical factors that might predict changes in QoL in those patients. Due to cultural variations and differing coping mechanisms, most HRQoL studies conducted in other nations may not accurately reflect the people in India. Only a few studies were available in the world literature that used the European Organization for Research and Treatment of Cancer (EORTC) Quality of Life Questionnaire (QLQ) C30 and QLQ HCC18 questionnaires to assess QoL data in HCC patients undergoing TACE [9,10]. A PubMed search turned up no Indian research on the health-related quality of life after TACE in patients with HCC. By identifying the factors contributing to poor HRQoL, it may be possible to implement targeted interventions to improve it.

## Materials and methods

Study population

This prospective single-center observational study was conducted in a tertiary care center in Kerala, South India, from January 2023 to March 2024. Forty-five patients with HCC (diagnosed by typical radiological criteria, tumor markers and/or histology who were selected for TACE according to standard criteria) who underwent TACE as the primary treatment modality (i.e., no other treatment taken previously {e.g., surgical resection, transplant, systemic chemotherapy, radioembolization, or chemoembolization}) were included in the study and followed up for three months. Patients with significant comorbid illnesses affecting quality of life and with a history or current extrahepatic malignancies were excluded from the study. Five patients were excluded from the final analysis (three patients were lost to follow-up, and two patients required alternative treatments). The demographic, clinical, laboratory, and radiological parameters were obtained during enrolment. The Child-Turcotte-Pugh (CTP) score, Model for End-Stage Liver Disease (MELD) score, BCLC stage, Albumin-Bilirubin (ALBI) grade, and Selection for Transarterial Chemoembolization Treatment (STATE) score were calculated to assess the severity of liver disease and the burden of HCC. The study was started only after getting approval from the local ethics committee (HEC no: 10/27/2021/MCT; dated December 30, 2021) and all participants' written informed consent.

Quality of life (QoL) data

HRQoL was assessed using Malayalam versions of the European Organization for Research and Treatment of Cancer (EORTC) QLQ C30 and HCC18 questionnaires (downloaded with permission for academic use from https://www.eortc.org/tools/) on the day before TACE and at two weeks and three months post-TACE. The first re-evaluation of HRQoL was planned two weeks after the treatment to negate the effects of post-embolization syndrome. The combination of questionnaires QLQ-C30 and HCC18 contains 48 questions, which can be grouped into 24 functional and 24 symptom scales (Table 1) [9,10]. The initial questionnaire includes six single-item scales of symptoms, five multi-item scales of functioning, three multi-item symptom scales, and one multi-item scale of global health. The supplementary HCC18 questionnaire comprises six multi-item scales and two single-item measures, addressing symptoms. Both QLQ-C30 and HCC18 are validated questionnaires [9,10]. The EORTC-published scoring manual was used to evaluate the HRQoL. A longitudinal transformation of the initial raw score was performed to facilitate better comparison. The questionnaires were administered by one of the authors and helped the patient and bystander fill out the questionnaires. The questionnaires were distributed just before the TACE session, after two weeks following the TACE session, and after three months, when the patient and bystander come for a visit to the hospital for follow-up. During the follow-up, the clinical and biochemical factors were also assessed.

**Table 1:** Quality of life instruments used in the study. QoL: quality of life

QoL instrument	Description
C30 summary score [9]	∑ (Physical functioning, role functioning, emotional functioning, cognitive functioning, social functioning, {100-fatigue}, {100-nausea and vomiting}, {100-pain}, {100-dyspnoea}, {100-insomnia}, {100-appetite loss}, {100-constipation}, {100-Diarrhea}) ÷ 13
C30 index score [9]	∑ ({100-physical functioning}, {100-role functioning}, {100-emotional functioning}, {100-cognitive functioning}, {100-social functioning}, {100-global QOL}, scores of fatigue, nausea and vomiting, pain, dyspnea, insomnia, appetite loss, constipation, diarrhea, financial difficulty) ÷ 15. Remarks: a lower score represents a less severe symptom/problem
HCC18 index score [10]	∑ (Scores of fatigue, body image, jaundice, nutrition, pain, fever, sex life, abdominal distension) ÷ 8. Remarks: a lower score represents a less severe symptom/problem.

Image acquisition and evaluation

Contrast-enhanced computed tomography was used as the baseline imaging, and MRI was employed for follow-up. All patients adhered to follow-up imaging, typically six weeks after the intervention. The response was evaluated using the modified RECIST (mRECIST) criteria. Then, patients were categorized into those with an objective response (CR+PR) and those with no response (SD+PD).

Interventional treatment

Conventional TACE was delivered to all patients. Transarterial access was achieved percutaneously via the common femoral artery, preferably on the right side, and a diagnostic catheter was threaded into the celiac trunk. Following this, the micro-catheter is advanced through a segmental or sub-segmental hepatic artery to a suitable position for embolization. Lipiodol is thoroughly mixed with chemotherapeutic drugs to form an emulsion, which is then carefully injected. Patients were given epirubicin suspended in 6 mL of Lipiodol (10 mg/mL) with a maximum dose of 60 mg of epirubicin per patient. Embolization was achieved using 1 or 2 mm of porous gelatin particles. Fluoroscopic guidance is obtained for this slow injection via the main feeding artery, allowing for uniform opacification of the entire tumor to be achieved. Stasis of antegrade flow of contrast or sub-stasis marks the completion of the procedure. TACE was performed by a single experienced interventional radiologist.

Data analysis

Quantitative variables were expressed in proportions and means, while proportions were used for qualitative variables. The association of the variables which were quantitative and not normally distributed in nature was analyzed using Mann-Whitney U test (for two groups) and Kruskal-Wallis test (for more than two groups), and variables that were quantitative and normally distributed in nature were analyzed using a paired t-test (for two groups) and ANOVA (for more than two groups), Wilcoxon signed-rank test was used for skewed distribution (for two groups), and Friedman test (for more than two groups). The correlation was tested using the Pearson correlation coefficient or Spearman’s rank correlation analysis. The association of the qualitative variables was analyzed using the chi-square test. A p-value of less than 0.05 will be considered statistically significant.

## Results

Baseline characteristics

The mean age was 61 years, with 18 (40%) individuals belonging to the 61-70-year age group. Thirty-four (77%) patients were males. The most common etiology was found to be metabolic-alcoholic liver disease (MetALD), also known as MASLD, and increased alcohol intake in 17 (37.8%) patients, followed by metabolic dysfunction-associated steatotic liver disease (MASLD) in 10 (22.2%) patients. Thirty-four (75.6%) patients belonged to CTP class A, and 11 (24.4%) were classified as CTP class B. Out of 45 patients, 24 (53.3%) were in BCLC stage A, while the remaining 21 (46.7%) were in BCLC B. Forty-two (93%) patients had a STATE score greater than 18. Thirty-six (80%) patients belonged to ALBI grade II. The size of the tumor was less than 2 cm in three patients (7%), 2-5 cm in 31 (69%), and more than 5 cm in 11 (24%) patients, with the mean size of the tumor being 4.26 cm. Only one focal lesion was present in 32 (72%) patients, and two focal lesions in 11 (24%) patients. One patient (2%) had three focal lesions, and another patient (2%) had four focal lesions. The mean MELD score was 11.8. There were no occurrences of serious complications apart from post-embolization syndrome after TACE. Seven patients (16%) had post-TACE syndrome.

QoL change

The QLQ-C30 and QLQ-HCC18 scales were analyzed to identify the mean scores before TACE, two weeks after TACE, and three months after TACE, as well as changes in scores and any associated significance. Overall, functional scales were found to be reduced at two weeks, although the reduction was statistically insignificant; however, a significant improvement was observed at three months. The Emotional Functioning Scale was the only exception to this. There was a general trend of decline in all functional and symptom scales at two weeks, followed by an improvement in all functional and symptom scales in both questionnaires by three months. The derivation of the C30 and HCC18 index scores encompassed all domains and items in the QLQ-C30 and QLQ-HCC18, respectively [9,10]. Absolute values improved at three months following the intervention, after a slight drop at two weeks (Table 2).

**Table 2:** Mean score in each domain of QoL in the QLQ-C30 and QLQ-HCC18 questionnaires in patients at baseline, two weeks and three months. GHS: global health status; PF: physical functioning; C30 SS: C30 summary score; QLQ: Quality of Life Questionnaire

Components	Baseline	Two weeks	Three months	Change (baseline vs. two weeks)	p-Value	Change (baseline vs. three months)	p-Value
Functional scales							
Global health status (GHS)	53.1±29.3	51.1±27.1	68±28.2	-2±23.1	0.455	14.9±32.5	0.045
Physical functioning (PF)	68.7±20.7	62±22.7	73.8±23.2	-6.7±18.9	0.005	5.1±23.5	0.158
Role functioning	71.1±25.4	62.2±26.3	75.2±27.6	-8.9±24.9	0.006	-4.1±27.6	0.790
Emotional functioning	73.1±30.1	68.3±25.7	79.8±25.7	-4.8±26.6	0.890	6.7±33.2	0.017
Cognitive functioning	82.2±23.3	76.7±24.3	83±20.9	-5.6±22.6	0.054	0.7±23.7	0.754
Social functioning	73.7±31.7	71.5±24.3	71.9±32	-2.2±36.9	0.789	1.8±26.2	0.261
C30 symptom scales							
Fatigue	46.4±32.5	51.6±30	30.5±28.2	-5.2±26	0.012	15.9±32.1	0.095
Nausea	12.6±24.1	17.4±16.3	8.5±19.6	-4.8±24.6	0.686	4.1±26.3	0.513
Pain	32.2±30.8	41.1±27.4	28.5±30.8	-8.9±26.1	0.005	3.7±26.8	0.712
Dyspnea	24.4±33	23±33	15.6±29.4	1.5±28.4	0.999	8.9±30.1	0.254
Insomnia	32.6±36.6	33.3±32.6	25.9±27.4	-0.7±37.3	0.896	6.7±36	0.220
Appetite	27.4±36.4	32.6±34.4	31.1±32.1	-5.2±31.7	0.279	-3.7±33.5	0.462
Constipation	24.4±31.3	25.2±33.2	14.8±27.2	-0.7±25.6	0.700	9.6±33.8	0.193
Diarrhea	8.1±19.6	3.7±22.8	5.9±26	4.4±22.7	0.280	2.2±26.1	0.850
Financial problems	63±37.1	66.7±36.9	68.9±34.4	-3.7±25.8	0.341	-5.9±28.7	0.173
HCC18 symptom scales							
Fatigue	39.5±27.5	48.1±29.5	31.9±26.8	-8.6±22.2	0.015	7.7±23.8	0.050
Body image	17±26	13.7±19.9	11.9±16.1	-3.3±18.0	0.221	5.2±19.4	0.080
Jaundice	19.3±24.6	18.9±21.7	14.8±18.1	0.4±18.1	0.784	4.4±23.1	0.242
Nutrition	28.4±23.4	31.1±20.7	22.7±21.9	-2.7±16.8	0.291	5.8±23.2	0.101
Pain	28.5±25.5	29.6±21.9	23.3±24.2	-1.1±23.4	0.752	5.2±31.7	0.279
Fever	27.8±33.1	25.2±28.6	22.2±27.5	2.6±20.7	0.406	5.6±28.6	0.200
Abdominal distension	21.5±37.5	19.3±32.6	15.6±22	2.2±31.2	0.430	5.9±31	0.067
Sexual dysfunction	10.4±19.9	16.3±33	14.1±29	-5.9±22.6	0.020	-3.7±17.3	0.051
Summary scores							
C30 SS	73.9	70.7	78.6	-3.2	0.159	4.7	0.082
C30 index	29.9	33.1	25.3	-4.2	0.084	4.6	0.359
HCC18 index	24	25.3	19.5	-1.3	0.54	4.5	0.061

Tumor response

The mRECIST criteria were applied for response evaluation at six weeks of intervention. Twenty-six patients (57.8%) achieved a complete response (CR), nine patients (20%) had a partial response (PR), three patients (6.7%) had stable disease (SD), and seven patients (15.6%) experienced progressive disease (PD).

Comparison of global health status, C30 summary score, C30 index, and HCC18 index with tumor response

The absolute values of global health status and QoL summary scores showed a positive response to TACE at three months in patients who had a complete response, partial response, and stable disease. In contrast, those with progressive disease experienced a deterioration in QoL across the timeline, although this was not statistically significant every time (Tables 3-6). This may be attributed to a lower sample size and greater variability among the population, as indicated by the standard deviations of each score.

**Table 3:** Comparison of GHS with tumor response (as assessed by mRECIST) at baseline, two weeks, and three months. GHS: global health status; mRECIST: modified Response Evaluation Criteria in Solid Tumors; CR: complete response; PR: partial response; SD: stable disease; PD: progressive disease

mRECIST	GHS (baseline)	GHS (two weeks)	GHS (three months)
CR	56.08±28.72	58.33±23.33	81.41±14.58
PR	57.40±21.01	52.77±26.34	66.66±17.17
SD	47.22±33.67	41.66±25.00	52.77±26.78
PD	39.28±32.53	26.18±18.27	26.18±21.74
P-value	0.52	0.02	<0.001

**Table 4:** Comparison of C30 SS with tumor response (as assessed by mRECIST) at baseline, two weeks, and three months. mRECIST: modified Response Evaluation Criteria in Solid Tumors; C30 SS: C30 summary score; CR: complete response; PR: partial response; SD: stable disease; PD: progressive disease

mRECIST	C30 SS (baseline)	C30 SS (two weeks)	C30 SS (three months)
CR	74.31±18.2	75.92±13.09	85.10±13.32
PR	73.03±23.38	63.80±20.28	73.67±15.33
SD	74.92±14.42	70.99±5.48	85.28±8.21
PD	73.00±17.46	59.85±15.55	57.99±16.63
P-value	0.98	0.04	<0.001

**Table 5:** Comparison of C30 index with tumor response (as assessed by mRECIST) at baseline, two weeks, and three months. mRECIST: modified Response Evaluation Criteria in Solid Tumors; CR: complete response; PR: partial response; SD: stable disease; PD: progressive disease

mRECIST	C30 index (baseline)	C30 index (two weeks)	C30 index (three months)
CR	29.62±18.53	27.91±13.69	18.67±13.12
PR	29.91±21.8	38.46±19.45	28.98±14.9
SD	29.69±15.58	33.46±8.13	20.34±8.9
PD	31.25±18.4	45.42±15.65	47.0±17.08
P-value	0.98	0.04	<0.001

**Table 6:** Comparison of HCC18 index with tumor response (as assessed by mRECIST) at baseline, two weeks, and three months. mRECIST: modified Response Evaluation Criteria in Solid Tumors; CR: complete response; PR: partial response; SD: stable disease; PD: progressive disease

mRECIST	HCC18 index (baseline)	HCC18 index (three weeks)	HCC18 index (three months)
CR	24.08	21.81	14.08
PR	25.12	28.92	25.58
SD	23.240	25.60	15.50
PD	22.89	33.33	33.78
P-value	0.98	0.33	0.01

Factors predicting change in global health status at three months after TACE

Higher AFP, CRP, and an increase in MELD and CTP scores were found to negatively affect the global health status at three months (Table 7). A younger age was associated with a better change in global health status (p=not significant). Higher albumin values correlated positively, indicating that individuals with higher albumin levels had a better overall health status. Among those with a negative correlation, the maximum was the MELD score (r=-0.84), suggesting that changes in HRQoL scores of HCC patients were more affected by the patient’s liver function status than by the presence of HCC.

**Table 7:** Factors predicting change in global health status at three months after TACE. TACE: transarterial chemoembolization; AFP: alpha-fetoprotein; CRP: C-reactive protein; CTP: Child-Turcotte-Pugh; MELD: Model for End-Stage Liver Disease

Variable	Pearson correlation coefficient	p-Value
AFP	-0.504	0.000
CRP	-0.563	0.000
CTP score at baseline	-0.103	0.50
CTP score at 3M	-0.707	0.000
Change in CTP score	-0.732	0.000
MELD score at baseline	0.055	0.719
MELD score at 3M	-0.526	0.000
Change in MELD score	-0.844	0.000
Tumor size	-0.214	0.15
Age	-0.099	0.51
Albumin	0.323	0.03

Global health status and QoL summary scores were inversely associated with tumor size and baseline AFP values. Factors predicting financial burdens, such as age, gender, marital status, occupation, and socioeconomic status, were not statistically significant. Upon comparing different QoL domains and summary scores, no statistically significant differences were found based on socioeconomic status, gender, or age.

## Discussion

TACE has been used to treat the intermediate stage of hepatocellular carcinoma for decades. By nature, TACE is palliative; therefore, it is crucial to focus on improving or maintaining health-related quality of life (HRQoL) and ensuring survival benefits, as well as imaging-based surrogate measures, in clinical research.

Our study demonstrated that neither the examined radiological parameters nor the clinical scores were suitable independent predictors for HRQoL post-TACE. Nevertheless, HRQoL evaluation has gained entry into existing guidelines and expert recommendations. In our study, the quality of life (QoL) initially decreased at two weeks but showed improvement by three months. This finding was comparable to that of Xie et al., which also reported lower QoL in the first month after the procedure but showed recovery by the third month [11]. A survey by Hassanin et al. also showed a steady improvement in QoL after TACE, which occurred later [12].

In our study, life-impairing symptoms, such as pain, nutritional problems, fatigue, or fever, showed a significant increase compared to the baseline pre-TACE evaluation, a finding similar to that observed by Hinrichs et al. [13]. The highest pre- and post-changes were observed in physical functioning, role functioning, pain, and fatigue at two weeks. The C30 summary score, C30 index, and HCC18 index also declined at two weeks, reflecting a net decline in quality of life in the immediate post-intervention period. This reduction in QoL at two weeks could be attributed to post-procedural pain, perceived deficiencies in their daily routines, and non-disclosure of the disease to the patient. The patient later learned the truth from others while admitted to the ward for TACE, causing acceptance difficulties and emotional distress.

Generally, the trend ameliorated in almost all symptoms and functional scales measured at three months. Exceptions to this were sex life, social functioning, appetite, and financial problems. Significant improvement was observed in global health status and emotional functioning at three months. These could be due to symptom improvement, objective tumor response, and fewer recurrences. This could also be attributed to patients' acceptance of the disease and support from family and healthcare workers.

Xie et al. reported a deteriorated quality of life (QoL) in the first month after the procedure, but it recovered by the third month, echoing the findings of our study [11]. However, a study by Hassanin et al. demonstrated a steady improvement in QoL after TACE [12]. The C30 summary score, C30 index, and HCC18 index showed an upward trend at three months, indicating an overall improvement in quality of life (QoL) in patients with hepatocellular carcinoma (HCC) who underwent transarterial chemoembolization (TACE) after three months.

A decreased quality of life (QoL) at two weeks, followed by later improvement at three months, may predict the need for extended symptomatic treatment with analgesic, antipyretic, and antiemetic medications, along with nutritional support, all of which are in addition to support from family and healthcare workers, to improve QoL [11].

The absolute values of the global health status score and C30 summary scores were better in responders and stable disease groups, whereas in the progressive disease group, they worsened. This is unlike the study by Hartrumpf et al., which demonstrated that QoL didn’t change across the mRECIST categories [14]. Studies by Qiao et al. have shown that quality of life (QoL) worsens as the tumor progresses, as observed in our study [15]. In our research, the quality of life (QoL) worsened and did not improve over time in patients with progressive disease.

Among the factors predicting change in global health status, a negative correlation was observed with the MELD score (r=-0.84), suggesting that the change in HRQoL scores of HCC patients was more affected by the patient’s liver function status than by the cancer.

Hartrumpf et al. showed that female gender and higher CRP at baseline were associated with a greater decrease in functional scales and an improvement in symptom scales [14]. In our study, CRP also had a predictive role in global health status. A lower benefit from TACE was noted in patients with high CRP values. Higher AFP values suggest higher tumor burden and higher biological aggressiveness, thereby leading to pain, fatigue, and anorexia. CRP also reflects systemic cachexia and malnutrition, which directly lead to muscle wasting, fatigue, malaise, reduced performance status, and reduced QoL [14].

QoL and liver function were prognostic factors for survival in HCC patients, regardless of the stage of the disease. Research conducted by Wang et al. revealed that the HRQoL scores of HCC patients were more influenced by the liver function level than the tumor itself [16]. Similarly, our study observed a correlation between MELD and QoL scores rather than tumor size or burden scores. These findings emphasize the importance of considering liver function and tumor burden when evaluating quality of life (QoL) in HCC patients.

Liver functional impairment in HCC patients can be attributed to various factors, such as viral infections, alcoholic hepatitis, extrahepatic infections, etc. Interventions to improve liver function, such as antiviral therapy, alcohol abstinence, improved nutrition, and treatment of infections, can potentially enhance patients' quality of life (QoL). Further studies are needed to determine whether treatments targeting liver function can improve the quality of life for patients with HCC.

QoL measurements play a crucial role in guiding clinical decision-making when selecting treatments. Insights from these valuable measurements can guide physicians in monitoring the severity of disease-related symptoms and the impact of treatment on a patient's overall well-being. When combined with other clinical data, such as tumor size and the presence of metastases, QoL data can assist physicians in determining the appropriate time to adjust dosages or treatment strategies.

Effective symptom management and coping strategies are essential for enhancing functioning in patients following TACE. Inadequate symptom management can result in feelings of hopelessness and a decline in QoL, potentially causing patients to discontinue treatment. Multidisciplinary teams comprising hepatologists, oncologists, dieticians, psychologists, palliative care specialists, and social workers can synergize to alleviate symptom burden, promote mental well-being, and restore physical functioning, thereby improving long-term satisfaction and treatment adherence. Psychological support and nutritional optimization are cornerstones of comprehensive care that significantly enhance post-TACE recovery, patient satisfaction, and overall well-being, thereby transforming the therapeutic landscape from disease-centered to truly patient-centered care.

Limitations of the study

This study was conducted on subjects from a single tertiary care referral center, with a follow-up period of three months. So, this may not be applicable to varied ethnic populations. Multicenter studies with bigger sample sizes and longer follow-up durations are needed. DEB-TACE was not offered in our institution. So, validating the above findings for DEB-TACE is also recommended. TACE can further exacerbate nutritional deficiencies due to reduced appetite, gastrointestinal symptoms, and catabolic stress. Nutritional support improves physical strength, immunity, and wound healing, directly influencing physical domains of HRQoL. Integrating nutritional and psychological support into routine post-TACE care adds a holistic approach to HCC management. However, we did not study the effect of nutritional or psychological support in improving the HRQoL.

## Conclusions

Transarterial chemoembolization (TACE) significantly improved global health status and summary scores at three months following intervention, except in patients with progressive disease. Two weeks after the intervention, a decrease in functional scales and worsening of symptom scales suggest the need for early post-TACE follow-up after discharge, addressing any symptoms promptly to improve quality of life in the initial days.

In our literature search, we evaluated the effect of TACE on HRQoL in the Indian population using the EORTC QLQ C30 and HCC18 questionnaires. Larger-scale studies using the same QoL questionnaires to assess the patients at multiple time points for a prolonged period are warranted. Newer scores that can predict QoL and response to treatment need to be studied and standardized to identify patients who may have favorable responses to TACE. Future trials are warranted to assess whether interventions aimed at enhancing nutritional status or providing psychological support can improve the quality of life of HCC patients.
